# Influence of Processing Parameters on Mechanical Properties and Degree of Crystallization of Polylactide

**DOI:** 10.3390/ma17143584

**Published:** 2024-07-19

**Authors:** Mariusz Fabijański, Tomasz Gołofit

**Affiliations:** 1 Plastics Processing Department, Faculty of Mechanical and Industrial Engineering, Warsaw University of Technology, 85 Narbutta Street, 02-524 Warsaw, Poland; 2 Department of High-Energetic Materials, Faculty of Chemistry, Warsaw University of Technology, 3 Noakowskiego Street, 00-664 Warsaw, Poland; tomasz.golofit@pw.edu.pl

**Keywords:** mechanical properties, degree of crystallization, polylactide, differential scanning calorimetry

## Abstract

This work attempts to assess the influence of process parameters on the change of mechanical properties and the degree of crystallinity of polylactide (PLA). PLA is a biodegradable material that has been widely used in various areas—from packaging, through medicine, to 3D printing, where it is used to produce prototypes. The method of processing is important, because the technological process and its parameters have a significant impact on the quality of the finished product. Their appropriate selection depends on quality and mechanical properties. The process parameters have an impact on the structure of PLA, specifically on the share of the crystalline phase, which is also important from the point of view of the functional properties of the finished product. This work assessed the impact of the technological parameters of the injection process on the final properties of the obtained samples. The obtained results of static tensile strength, hardness and differential scanning calorimetry (DSC) analysis confirm that changing these parameters affects the material properties.

## 1. Introduction

The widespread use of plastics has resulted in a negative impact on the environment. These include long decomposition times, lingering in landfills and a significant carbon footprint. On the one hand, polymeric materials have become synonymous with progress, but on the other hand, they have become a global problem. One of the activities limiting these negative impacts is the development of biodegradable polymers that decompose in the natural environment. Biodegradable polymers are an alternative to traditional plastics.

A representative of this group is polylactide (PLA). It is a thermoplastic material that is completely biodegradable within six months to two years. These include aliphatic polyesters. Its good mechanical and processing properties enable it to be formed using basic methods such as injection, extrusion, thermoforming and 3D printing [1,2,3,4,5].

PLA, due to its thermoplastic and processing capabilities on typical machines, as well as good mechanical properties, biodegradability, and non-toxicity of both the material itself and its decomposition products, has a very wide range of applications, from packaging to medicine. It is used as a biodegradable material for bioresorbable surgical threads, clips, staples, and surgical meshes, as well as for bone fixation screws and capsules that release a predetermined dose of the drug at a specific time. Products made of PLA also include membranes that accelerate the healing of extensive wounds, hygiene products, surgical masks, and medical clothing [6,7,8,9,10,11].

Other applications comprise packaging such as tapes, labels, waste bags, and beverage bottles, along with food and cosmetic packaging [12,13,14,15,16,17,18,19,20].

In agriculture, it is used for protective foils when growing plants, for tunnels to protect flowers and vegetables, and to protect young trees against pests and negative temperatures. After the end of their useful life, these products remain in the soil and biodegrade, thus enriching it further. PLA is also used as input material for 3D printers. It is expected that the scope of use of PLA will systematically expand [21,22,23,24,25].

Biodegradation itself is a process in which the polymer material decomposes under the influence of environmental factors. This process is favored by appropriate ambient humidity and temperature as well as living organisms such as yeast, bacteria and fungi that are found in the surroundings of the material [26,27]. The polymer material may undergo complete biodegradation with the release of e.g., carbon dioxide, ammonia, methane or water, or only partial biodegradation, for instance of one of the components of the material [28,29,30,31,32,33].

However, the rate of biodegradation, in addition to humidity and heat, is determined by the shape of the object, its geometry and thickness. Low crystallinity, low molecular weight, chemical groups susceptible to the action of appropriate enzymes, and water absorption by the polymer are important factors. PLA naturally undergoes a crystallization process, and, in this respect, it is like PET, but the crystalline phase is very poorly biodegradable [34,35,36,37].

The degree of crystallinity of PLA can be very high, up to 60%. The melting point of this material ranges from 170 to 180 °C, and the glass transition temperature is approximately 65 °C. It crystallizes fastest at 110 °C. It is a relatively high-density material, ranging from 1.2 g/cm^3^ to 1.3 g/cm^3^. It is possible to control the share of the crystalline phase through appropriate machine settings, and thus, the time and speed of PLA decomposition can be adjusted [38,39,40]. Control of the share of the crystalline phase in PLA can be achieved by regulating the parameters of the production and technological process, such as melting temperature, cooling speed, and processing time. Additives to PLA may also influence the share of the crystalline phase. Therefore, by adjusting these parameters, it is possible to regulate the time and speed of PLA decomposition by controlling the share of the crystalline phase. However, this process requires a thorough understanding of the PLA crystallization mechanisms and adaptation of the process conditions to specific application requirements [41,42,43,44,45].

PLA can exist in various isometric (or polymorphic) forms that differ in their crystal system and physical properties. Isomerization refers to the change from one crystalline form to another, which can be triggered by various factors such as temperature, time, pressure, processing method, etc. PLA can crystallize in several different forms, including α (alpha), β (beta) and γ (gamma). Each of these forms has a different crystal system and properties. α-form (alpha): This is the most stable and common crystalline form of PLA. It is characterized by an orthorhombic system. β (beta) form: This is less common and can be induced under certain processing conditions such as high temperature stretching. γ (gamma) form: This is rare and occurs under specific processing conditions. Each isometric form of PLA has different mechanical and thermal properties. For example, the α form tends to have a higher melting point and greater thermal stability than other forms. The β form may have increased mechanical strength in the tensile direction [30,31,32,33,34,35,41,42,43,44,45].

Processing conditions such as cooling rate, crystallization temperature, and processing time can influence the formation of specific isometric forms of PLA [44,46,47,48,49].

The aim of this work is to carry out the sample injection process with various machine settings (temperature, pressure, speed) and to assess the impact of these parameters on the mechanical properties and degree of crystallization of PLA.

This research will make it possible to attempt to explain the phenomenon of PLA crystallization in the injection molding process. It is also an attempt to control the degree of crystallinity and mechanical properties of the product by selecting appropriate technological parameters of the manufacturing process. The innovativeness of the work lies in a holistic approach to the analysis of processing processes and their impact on the properties of PLA products. This technological and process approach can bring significant benefits to the production of products with the desired characteristics.

To confirm the thesis stated in the introduction, this study used tests to determine strength in a tangential test and hardness, as critical parameters for this material, to assess the mechanical properties. The degree of crystallinity was determined based on the obtained differential scanning calorimetry (DSC) curves.

## 2. Materials and Methods

In this study, polylactide (PLA) from NatureWorks (Minneapolis, MN, USA) under the name Ingeo Biopolymer 3251D was used for research; it is intended for processing using injection technology. Table 1 provides data for the polylactide Ingeo Biopolymer 3251D [46].

To prepare samples for testing, a UT90 horizontal screw injection molding machine from Ponar Żywiec (Żywiec, Poland) of the UT series for thermoplastics was used, with a five-point, double, lever mold closing system and direct drive of the screw with a high-torque hydraulic motor. Peripheral devices used in the process include an injection mold with replaceable inserts for paddles and bars, a thermostat used to maintain a constant temperature of the injection mold, a DARwag electronic scale, and a KC 100/200 dryer.

The parameters of the paddle injection technological process are given in Table 2, where the temperature of the technological process has changed. In this way, three groups of material with different processing temperatures were obtained: 180 °C, 195 °C, 210 °C.

However, the change in the injection pressure and mold temperature settings for the preparation of the bars and the assessment of the change in the impact of these settings on the change in the degree of crystallinity determined in the DSC tests are given in Table 3. To properly prepare the material for testing, PLA was dried before the processing process to remove moisture, as it could have a negative impact on the technological process and the quality of the obtained products. The treatment was carried out for 8 h at a temperature of 80 °C in accordance with the material manufacturer’s recommendations.

For mechanical tests, three series of samples in the form of paddles were made changing the processing temperature according to the parameters included in Table 2. However, samples in the form of bars for DSC tests were made in six series, changing the mold temperature and injection pressure in accordance with Table 3. In order to check the injection process, mass monitoring was carried out, and each time the samples were weighed after the injection process. Table 4 shows the average weight of the injected samples.

A small standard deviation indicates that the technological process was stable, and the obtained injection mass values are closely concentrated around the average mass value of the tested samples.

Before commencing the actual tests, all samples were air-conditioned at a constant temperature of 23 °C and 50% humidity for 120 h. The examination of strength characteristics in a static tensile test was carried out in accordance with the guidelines contained in the ISO 527-1 [50] and ISO 527-2 [51] on the Fu1000e testing machine from Heckert (Chemnitz, Germany) with a measuring head up to 10 kN. The measurement consisted of static stretching of standardized samples at a constant speed of 2 mm/min. During the test, the change in force and elongation at break were recorded. At least five repetitions were performed for each series so that the measurement error was within 20%. Samples that broke in the machine holders or outside the extensometer were discarded.

Hardness was determined using the ball indentation method in accordance with the ISO 2039-1 standard [52] on a 101 Kudi Gnehm plastic hardness tester (Basel, Switzerland). Ten measurements were made on each batch of samples, and then the average hardness value was determined, and the measurement uncertainty was estimated in accordance with the procedure adopted in the standard.

DSC tests were carried out using a DSC Q2000 differential scanning calorimeter (TA Instruments, New Castle, DE, USA) in accordance with ISO 11357-1 [53]. The device was connected to a computer equipped with Q Series software (https://www.tainstruments.com/support/software-downloads-support/downloads/, accessed on 17 June 2024) for processing data obtained during measurements.

DSC studies were performed using a Q2000 flow calorimeter (TA Instruments, New Castle, DE, USA). Indium was used for calibration as a standard substance. Measurements were carried out at temperatures from −10 to 250 °C, with a constant temperature increase of 10 °C/min, in a nitrogen flow of 50 mL s^−1^. The system was heated, cooled, and heated again.

Then, the calorimeter was programmed by entering the required data into the program, such as sample weight, maximum heating and minimum cooling temperatures, and temperature jump (every 10 °C/min). Two measurements were made on one sample. First, the sample was quickly cooled to −10 °C, then heated, cooled and heated again. This treatment allows you to get rid of the stresses left in the sample after the processing process. The entire measurement took about an hour and a half. The process proceeded according to the following scheme:

1. Stabilization at −10.00 °C,
2. Heating 10.00 °C/min to 220.00 °C,
3. Marking the end of cycle 1,
4. Data recording,
5. Heating 10.00 °C/min to −10.00 °C,
6. Stabilization at −10.00 °C,
7. Data recording,
8. Heating 10.00 °C/min to 220.00 °C,
9. End of measurement.

From each series of samples produced with the given technological parameters, three samples were taken and tested. In this way, DSC thermograms were obtained for six samples for which the technological parameters were changed.

## 3. Results and Discussion

Table 5 summarizes the average results obtained in the static tensile test. Strength and elongation at break were analyzed. The obtained results show that a change in the processing (injection) temperature influences the change in stress and strain at break. It is not clearly large, but observable. The course of all stretching curves is similar, and straight. After reaching the maximum stress, the samples experienced brittle fracture.

The hardness measurement results are presented in Table 6. The values given are averages from a series of measurements. As the process temperature increases, a slight decrease in the hardness of the material is observed. The processing temperature may affect the rheology of the material, i.e., its behavior during the process. These changes may lead to micro and macro changes in the structure, which in turn may affect its hardness. The obtained values, similarly to the results obtained in the static tensile test, oscillate within the error limit. However, this does not exclude the fact that a slight decrease in hardness was observed.

**Figure 1:**
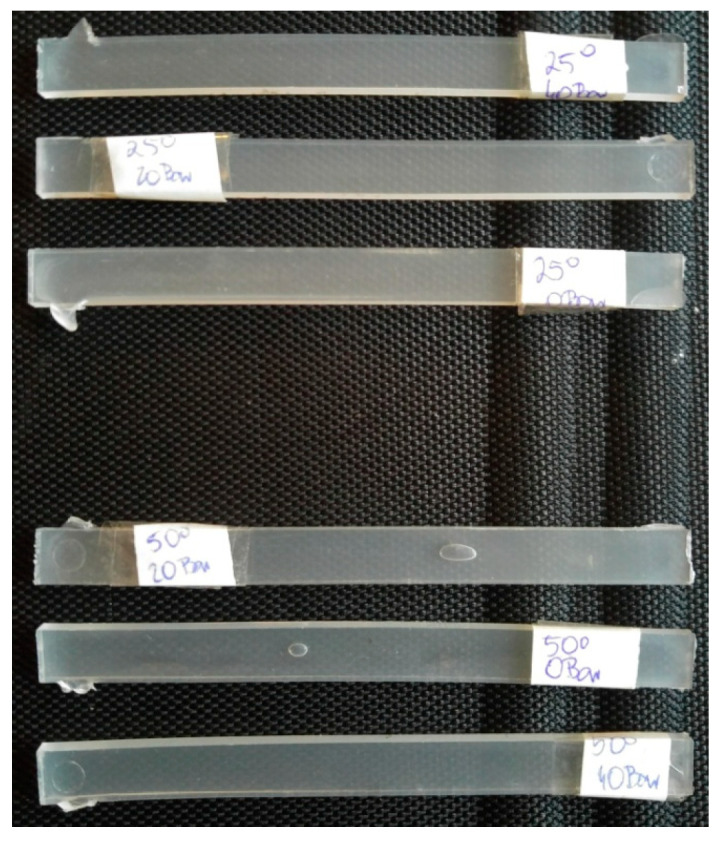
View of samples for DSC testing.

In order to prepare samples for determining the degree of crystallinity, a fragment of previously produced “bars” (Figure 1) weighing approximately 5 mg was taken.

**Figure 2:**
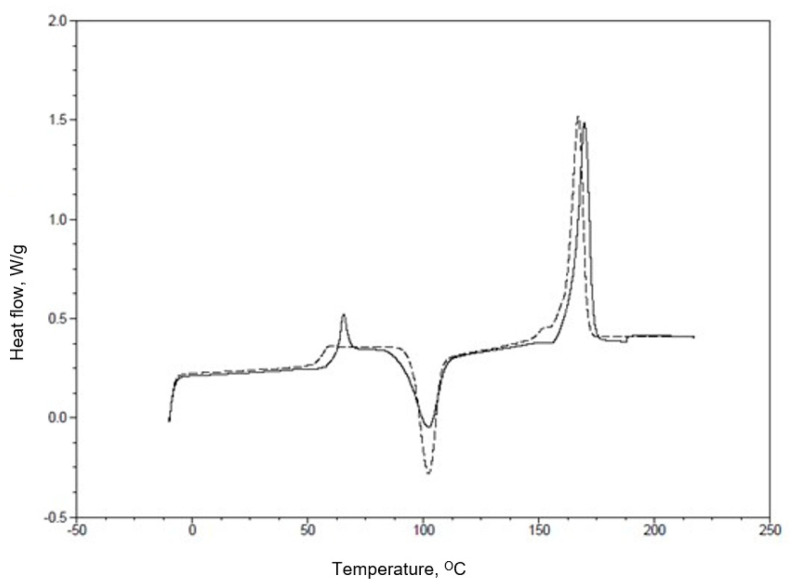
Example of a DSC thermogram for the PLA25C0B sample with two measurements, dashed line—first heating process, solid line—second heating.

In this way, DSC thermograms (a graph of temperature versus heat flow) were obtained for six samples. All calculations were performed on the first measurements. The analyzed process curve on the graphs obtained from the device was marked as a solid line, as shown in Figure 2 for the sample marked PLA25C0B (Table 3).

DSC measurements of the obtained samples were performed. Examples of DSC curves obtained for the PLA25C0B sample are shown in Figure 2.

The DSC curves presented in Figure 2 show the changes that the PLA samples underwent during the measurements. The first, low-temperature transformation is the process of glassing the sample, which is associated with a step change in heat capacity. In the first measurement, this transformation is superimposed by the B-relaxation process. For the second heating cycle, B-relaxation is not visible. At a temperature of approximately 100 °C, an exothermic transformation is visible, associated with the crystallization of the amorphous phase present in the sample. The last high-temperature transformation visible on the DSC curve is the melting of the crystalline phase. The obtained DSC results are presented in Table 7.

In the program (TA Universal Analysis), on ready-made thermographs, calculations were made of the increase in heat capacity in the process of glass transition, crystallization, and melting, as well as calculations of extrapolated temperatures (onset temperatures) of these transitions. The onset temperature was determined at the intersection of the tangent edge of the peak and the baseline.

**Figure 3:**
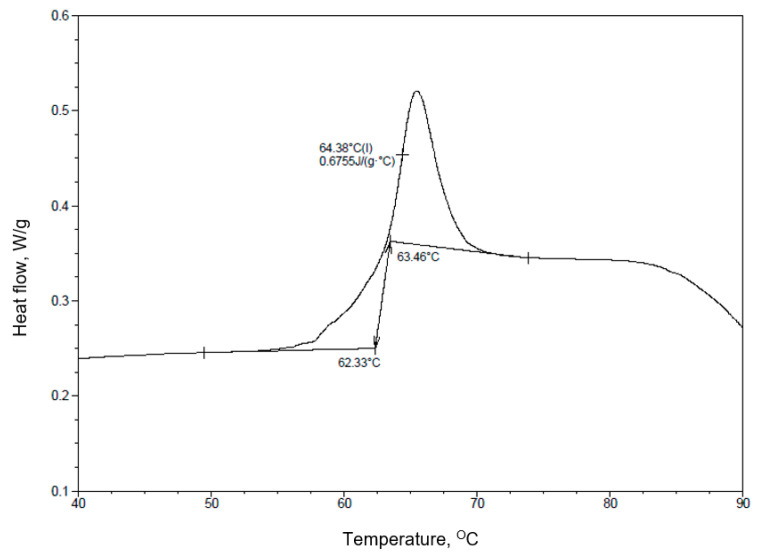
Glass transition curve for PLA25C0B (Table 3). The arrows indicate the beginning and end of the measurement section.

The first transformation is the glass transition process (Figure 3). To determine the desired values, the baseline lines before and after the glass transition process were marked on the graph. After determining the beginning and end points of the process, the end temperature of the transformation (63.46 °C), the onset temperature (64.38 °C) and the change in heat capacity (0.6755 J/g * °C) were determined.

**Figure 4:**
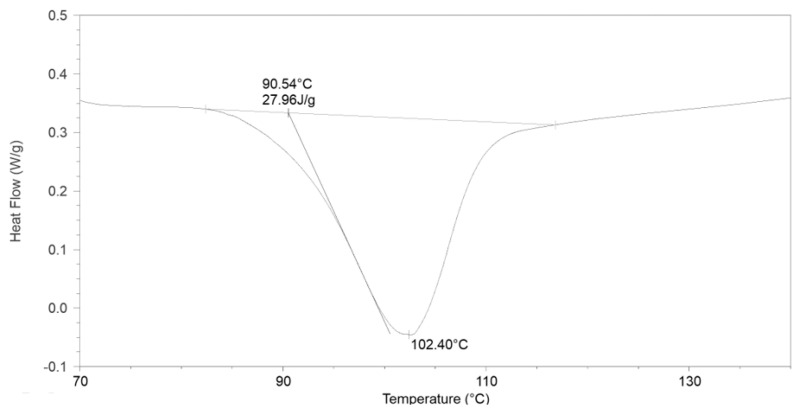
The course of the crystallization process for the PLA25C0B sample (Table 3).

The second transformation is crystallization (Figure 4). Characteristic parameters are determined in the same way as for glass transition. The final transformation temperature is 102.40 °C, the final temperature is 90.53 °C, and the change in heat capacity is 27.97 J/g.

**Figure 5:**
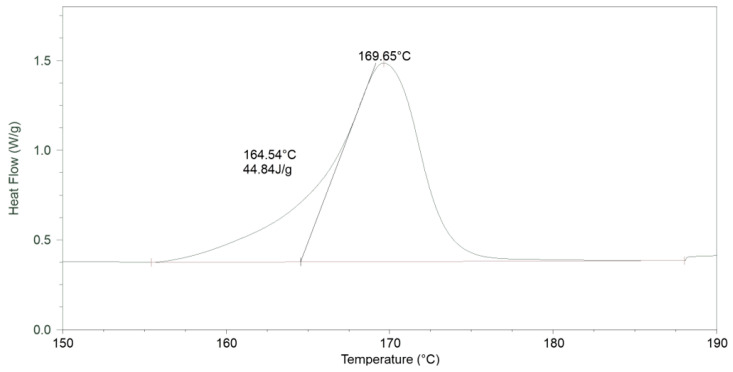
Melting process for PLA25C0B (Table 3).

The last transformation to take place was the melting process (Figure 5). In this case, the characteristic parameters were also determined in the same way as in the case of glass transition. The final transformation temperature was 169.65 °C, while the final temperature was 164.58 °C, and the change in capacity was 43.30 J/g.

All thermograms for all samples included in Table 3 were analyzed in the same way. Then, the degree of crystallinity of individual PLA samples was determined and the impact of changing process parameters (Table 3) on this degree was determined.

The degree of crystallinity of the polylactide samples was calculated based on their enthalpy of melting. The determined melting enthalpy should be corrected for the cold crystallization process visible on the DSC curve. The degree of crystallinity of polylactide was determined using Formula (1).

(1) $$\alpha =\frac{∆H_{t}-∆H_{k}}{∆H_{t100\%}}\cdot 100\%$$

where Δ*H_t_*—fusion enthalpy; Δ*H_t_*—crystallization enthalpy; Δ*H_t_*_100%_—enthalpy of the melting process of a 100% crystalline sample of polylactide.

The enthalpy of the melting process of a 100% crystalline sample of polylactide is 93 J/g [22]. The summary of DSC results is presented in Table 7, while the calculated degree of crystallinity of the tested samples is given in Table 8.

From the analysis of the collected results, it can be concluded that the degree of crystallinity changes with increasing mold temperature and pressure. One of the conclusions is the proposal to conduct tests at higher temperatures and longer sample cooling times. Then these changes would probably be more pronounced.

Based on the summary of the thermal capacity jump (Table 7) for individual samples, these measurements are very similar to each other. In the melting process, the range between measurements is high. However, in the crystallization process, the increase in heat capacity rises slightly with increasing temperature and pressure. At the same time, as the mold temperature and injection pressure increase, the enthalpy of crystallization increases slightly. This confirms the thesis that the degree of crystallization of PLA depends on the process conditions.

## 4. Conclusions

The results obtained from tensile strength, hardness and differential scanning calorimetry (DSC) tests of PLA confirm that the technological processing process affects the properties and structure of the material. The adopted technological assumptions of the injection process showed differences in mechanical properties and the degree of crystallization. Although these differences may not be so spectacular, they confirm the thesis put forward in this work. This means that even minor changes in the technological processing process may affect the final properties of the material. Further research and experimentation can help understand these relationships and optimize the process to obtain the desired material properties.

The obtained results also suggest that further research in this area is justified and may bring valuable results leading to the improvement of production processes in plastics processing. Extending the research with microscopic structural analyses and detailed studies of the PLA crystal phases could further enrich the knowledge about the impact of processing parameters on the final properties of products.

## Figures and Tables

**Table 1:** Properties of Ingeo Biopolymer 3251D [46].

Parameters	Unit	Value
Specific gravity	N/m^3^	1.24
Mass flow rate index (MFR) 210 °C; 2.16 kg	g/10 min	70–85
Crystallization temperature	°C	160–170
Glass transition temperature	°C	55–65
Transparency	-	transparent
Tensile strength	MPa	48
Tensile elongation	%	2.5
Impact strength by Izod test	J/m	16
Flexural strength	MPa	83
Processing parameters		
Injection: temperature	°C	180–205
Injection: mold temperature	°C	25

**Table 2:** Parameters of the injection process of samples for strength and hardness tests.

Injection Parameters	Unit	Value		
Injection				
speed	%	40		
pressure	bar	80		
Clamping				
time	s	10		
clamping pressure	bar	60		
Closing force				
average	N	887		
Closing the mold				
pressure	bar	130		
speed	%	40		
mold protection time	s	5		
cycle time	s	120		
backpressure	bar	5		
Opening the mold				
backpressure	bar	10		
cooling time	s	15		
temperature	°C	25		
Temperature				
		PLA-I	PLA-II	PLA-III
Nozzle		180 ± 2	195 ± 2	210 ± 2
Zone 1	°C	180 ± 2	195 ± 2	210 ± 2
Zone 2		180 ± 2	195 ± 2	210 ± 2
Zone 3		180 ± 2	195 ± 2	210 ± 2

**Table 3:** Values of injection pressure and mold temperatures when preparing samples for testing to determine the degree of crystallinity.

Sample Identifiers	Mold Temperature, °C	Injection Pressure, Bar
PLA25C0B	25 ± 2	10 ± 1
PLA25C20B	25 ± 2	20 ± 1
PLA25C40B	25 ± 2	40 ± 2
PLA50C0B	50 ± 2	10 ± 1
PLA50C20B	50 ± 2	20 ± 2
PLA50C40B	50 ± 2	40 ± 2

**Table 4:** Average sample injection weight depending on the process temperature.

	PLA-I—180 °C	PLA-II—195 °C	PLA-III—210 °C
Average injection weight, g	24.74	24.47	24.16
Standard deviation	0.016	0.047	0.062

**Table 5:** Strength and elongation at break depending on the processing temperature.

Sample Identifiers	Processing Temperature, °C	Tension, MPa	Elongation at Break, %
PLA-I	180	70.8 ± 3.4	9.97 ± 0.48
PLA-II	195	70.4 ± 3.4	9.77 ± 0.47
PLA-III	210	70.7 ± 3.4	10.01 ± 0.48

**Table 6:** Average hardness of samples depending on the processing temperature.

	PLA-I—180 °C	PLA-II—195 °C	PLA-III—210 °C
Hardness, MPa	128.8 ± 5.2	121.5 ± 6.0	117.1 ± 7.2

**Table 7:** Summary of DSC measurement results for individual samples.

	The Glass Transition Process			Crystallization Process			Melting Process		
	Final Temp., °C	Onset Temp., °C	Change in Heat Capacity, J/(g * °C)	Final Temp., °C	Onset Temp., °C	Crystallisation Enthalpy, J/g	Final Temp., °C	Onset Temp., °C	Melting Enthalpy, J/g
PLA25C0B	63.5	64.4	0.68	102.4	90.5	27.97	169.7	164.6	43.30
PLA25C20B	63.9	64.8	0.76	99.5	89.7	28.99	169.3	164.8	43.14
PLA25C40B	63.8	64.8	0.63	101.2	91.8	29.12	170.1	165.1	43.94
PLA50C0B	64.1	65.1	0.58	103.8	92.5	29.51	170.9	162.7	43.33
PLA50C20B	63.9	65.1	0.71	102.6	92.3	29.95	169.4	162.6	43.28
PLA50C40B	65.4	66.2	0.66	102.0	93.5	31.03	169.6	163.5	43.71
min	63.5	64.4	0.58	12.4	89.7	27.97	169.3	162.6	43.14
max	65.4	66.2	0.76	103.8	93.5	31.03	170.9	165.1	43.94
difference	1.9	1.8	0.18	4.3	3.8	3.06	1.6	2.5	0.80

**Table 8:** Calculated average degree of crystallinity of individual samples.

	The Degree of Crystallinity, %
PLA25COB	16.5
PLA25C20B	15.2
PLA25C40B	15.9
PLA50C0B	14.9
PLA50C20B	14.3
PLA50C40B	13.6

## Data Availability

All data are provided in the manuscript.

## References

[1] Yusuf M.O., Johari M.A.M., Ahmad Z.A., Maslehuddin M. (2014). Strength and microstructure of alkali-activated binary blended binder containing palm oil fuel ash and ground blast-furnace slag. Constr. Build. Mater..

[2] Part W.K., Ramli M., Cheah C.B. (2015). An overview on the influence of various factors on the properties of geopolymer concrete derived from industrial by-products. Constr. Build. Mater..

[3] McLellan B.C., Williams R.P., Lay J., van Riessen A., Corder G.D. (2011). Costs and carbon emissions for geopolymer pastes in comparison to ordinary portland cement. J. Clean. Prod..

[4] Biricik H., Kırgız M.S., Galdino A.G.d.S., Kenai S., Mirza J., Kinuthia J., Ashteyat A., Khitab A., Khatib J. (2021). Activation of slag through a combination of NaOH/NaS alkali for transforming it into geopolymer slag binder mortar—Assessment the effects of two different Blaine fines and three different curing conditions. J. Mater. Res. Technol..

[5] Zhuang X.Y., Chen L., Komarneni S., Zhou C.H., Tong D.S., Yang H.M., Yu W.H., Wang H. (2016). Fly ash-based geopolymer: Clean production, properties and applications. J. Clean. Prod..

[6] Caldas P.H.C.H., de Azevedo A.R.G., Marvila M.T. (2023). Silica fume activated by NaOH and KOH in cement mortars: Rheological and mechanical study. Constr. Build. Mater..

[7] Marvila M.T., de Azevedo A.R.G., Júnior J.A.T.L., Vieira C.M.F. (2023). Activated alkali cement based on blast furnace slag: Effect of curing type and concentration of Na_2_O. J. Mater. Res. Technol..

[8] Cong X., Zhou W., Elchalakani M. (2020). Experimental study on the engineering properties of alkali-activated GGBFS/FA concrete and constitutive models for performance prediction. Constr. Build. Mater..

[9] Le H.-B., Bui Q.-B., Tang L. (2021). Geopolymer Recycled Aggregate Concrete: From Experiments to Empirical Models. Materials.

[10] Zhang H., Li L., Yuan C., Wang Q., Sarker P.K., Shi X. (2020). Deterioration of ambient-cured and heat-cured fly ash geopolymer concrete by high temperature exposure and prediction of its residual compressive strength. Constr. Build. Mater..

[11] Thomas R.J., Peethamparan S. (2015). Alkali-activated concrete: Engineering properties and stress–strain behavior. Constr. Build. Mater..

[12] Amlashi A.T., Abdollahi S.M., Goodarzi S., Ghanizadeh A.R. (2019). Soft computing based formulations for slump, compressive strength, and elastic modulus of bentonite plastic concrete. J. Clean. Prod..

[13] Zhao H., Li S., Zang X., Liu X., Zhang L., Ren J. (2023). Uncertainty quantification of inverse analysis for geomaterials using probabilistic programming. J. Rock Mech. Geotech. Eng..

[14] Ahmad A., Chaiyasarn K., Farooq F., Ahmad W., Suparp S., Aslam F. (2021). Compressive strength prediction via Gene Expression Programming (GEP) and Artificial Neural Network (ANN) for concrete containing RCA. Buildings.

[15] Song H., Ahmad A., Ostrowski K.A., Dudek M. (2021). Analyzing the compressive strength of ceramic waste-based concrete using experiment and Artificial Neural Network (ANN) approach. Materials.

[16] Li Q., Song Z. (2023). Prediction of compressive strength of rice husk ash concrete based on stacking ensemble learning model. J. Clean. Prod..

[17] Mansouri E., Manfredi M., Hu J.-W. (2022). Environmentally friendly concrete compressive strength prediction using hybrid machine learning. Sustainability.

[18] Fang J., Xie M., He X., Zhang J., Hu J., Chen Y., Yang Y., Jin Q. (2022). Machine learning accelerates the materials discovery. Mater. Today Commun..

[19] Arrieta A.B., Díaz-Rodríguez N., Del Ser J., Bennetot A., Tabik S., Barbado A., Garcia S., Gil-Lopez S., Molina D., Benjamins R. (2020). Explainable Artificial Intelligence (XAI): Concepts, taxonomies, opportunities and challenges toward responsible AI. Inf. Fusion.

[20] Naser M. (2021). An engineer’s guide to eXplainable Artificial Intelligence and Interpretable Machine Learning: Navigating causality, forced goodness, and the false perception of inference. Autom. Constr..

[21] Friedman J.H. (2001). Greedy function approximation: A gradient boosting machine. Ann. Stat..

[22] Pedregosa F., Varoquaux G., Gramfort A., Michel V., Thirion B., Grisel O., Blondel M., Prettenhofer P., Weiss R., Dubourg V. (2011). Scikit-learn: Machine Learning in Python. J. Mach. Learn. Res..

[23] Lundberg S.M., Lee S. A unified approach to interpreting model predictions. Proceedings of the 31st International Conference on Neural Information Processing Systems.

[24] Zhao H., Li M., Zhang L., Zhao L., Zang X., Liu X., Ren J. (2024). Multi-objective optimization for composition design of civil materials based on data-driven method. Mater. Today Commun..

[25] Steinerova M. (2011). Mechanical properties of geopolymer mortars in relation to their porous structure. Ceram. Silik..

[26] Provis J.L., Duxson P., van Deventer J.S. (2010). The role of particle technology in developing sustainable construction materials. Adv. Powder Technol..

[27] Van Deventer J.S., Provis J.L., Duxson P. (2011). Technical and commercial progress in the adoption of geopolymer cement. Miner. Eng..

[28] Bagheri A., Nazari A. (2014). Compressive strength of high strength class C fly ash-based geopolymers with reactive granulated blast furnace slag aggregates designed by Taguchi method. Mater. Des..

[29] Fernández-Jiménez A., Palomo A., López-Hombrados C. (2006). Engineering properties of alkali-activated fly ash. ACI Mater. J..

[30] Wang S., Xia P., Chen K., Gong F., Wang H., Wang Q., Zhao Y., Jin W. (2023). Prediction and optimization model of sustainable concrete properties using machine learning, deep learning and swarm intelligence: A review. J. Build. Eng..

[31] Ma G., Cui A., Huang Y., Dong W. (2022). A Data-Driven Influential Factor Analysis Method for Fly Ash–Based Geopolymer Using Optimized Machine-Learning Algorithms. J. Mater. Civ. Eng..

[32] Lahoti M., Narang P., Tan K.H., Yang E.-H. (2017). Mix design factors and strength prediction of metakaolin-based geopolymer. Ceram. Int..

[33] Gunasekara C., Atzarakis P., Lokuge W., Law D.W., Setunge S. (2021). Novel Analytical Method for Mix Design and Performance Prediction of High Calcium Fly Ash Geopolymer Concrete. Polymers.

[34] Zhao T., Wu H., Sun J., Wen X., Zhang J., Zeng W., Shen H., Hu Z., Huang P. (2022). Immobilization of uranium tailings by phos-phoric acid-based geopolymer with optimization of machine learning. J. Radioanal. Nucl. Chem..

[35] Huang Y., Huo Z., Ma G., Zhang L., Wang F., Zhang J. (2023). Multi-objective optimization of fly ash-slag based geopolymer considering strength, cost and CO2 emission: A new framework based on tree-based ensemble models and NSGA-II. J. Build. Eng..

[36] Wang Q., Hussain A., Farooqi M.U., Deifalla A.F. (2022). Artificial intelligence-based estimation of ultra-high-strength concrete’s flexural property. Case Stud. Constr. Mater..

[37] Li Y., Shen J., Lin H., Li Y. (2023). Optimization design for alkali-activated slag-fly ash geopolymer concrete based on artificial intelligence considering compressive strength, cost, and carbon emission. J. Build. Eng..

[38] Atiş C.D., Bilim C., Çelik Ö., Karahan O. (2009). Influence of activator on the strength and drying shrinkage of alkali-activated slag mortar. Constr. Build. Mater..

[39] Krizan D., Zivanovic B. (2002). Effects of dosage and modulus of water glass on early hydration of alkali–slag cements. Cem. Concr. Res..

[40] He J., Bai W., Zheng W., He J., Sang G. (2021). Influence of hydrated lime on mechanical and shrinkage properties of alkali-activated slag cement. Constr. Build. Mater..

[41] Prokhorenkova L., Guaev G., Vorobev A., Dorogush A.V., Gulin A. CatBoost: Unbiased boosting with categorical features. Proceedings of the 32nd International Conference on Neural Information Processing Systems.

[42] Wang Y., Huang X., Ren X., Chai Z., Chen X. (2022). In-process belt-image-based material removal rate monitoring for abrasive belt grinding using CatBoost algorithm. Int. J. Adv. Manuf. Technol..

[43] Lee S., Vo T.P., Thai H.-T., Lee J., Patel V. (2021). Strength prediction of concrete-filled steel tubular columns using Categorical Gradient Boosting algorithm. Eng. Struct..

[44] Endres S.C., Sandrock C., Focke W.W. (2018). A simplicial homology algorithm for Lipschitz optimization. J. Glob. Optim..

[45] Wu Y., Zhou Y. (2022). Hybrid machine learning model and Shapley additive explanations for compressive strength of sustainable concrete. Constr. Build. Mater..

[46] Mangalathu S., Hwang S.-H., Jeon J.-S. (2020). Failure mode and effects analysis of RC members based on machine-learning-based SHapley Additive exPlanations (SHAP) approach. Eng. Struct..

[47] Ogami C., Tsuji Y., Seki H., Kawano H., To H., Matsumoto Y., Hosono H. (2021). An artificial neural network–pharmacokinetic model and its interpretation using Shapley additive explanations. CPT Pharmacomet. Syst. Pharmacol..

[48] Nguyen N.-H., Tong K.T., Lee S., Karamanli A., Vo T.P. (2022). Prediction compressive strength of cement-based mortar containing metakaolin using explainable Categorical Gradient Boosting model. Eng. Struct..

[49] Ravikumar D., Neithalath N. (2012). Effects of activator characteristics on the reaction product formation in slag binders activated using alkali silicate powder and NaOH. Cem. Concr. Compos..

[50] (2012). Plastics—Determination of Tensile Properties—Part 1: General Principles.

[51] (2012). Plastics—Determination of Tensile properties—Part 2: Test Conditions for Moulding and Extrusion Plastics.

[52] (2001). Plastics—Determination of Hardness—Part 1: Ball Indentation Method.

[53] (2016). Plastics—Differential Scanning Calorimetry (DSC)—Part 1: General Principles.

